# Facilitation among plants in alpine environments in the face of climate change

**DOI:** 10.3389/fpls.2014.00387

**Published:** 2014-08-12

**Authors:** Fabien Anthelme, Lohengrin A. Cavieres, Olivier Dangles

**Affiliations:** ^1^Institut de Recherche Pour le Développement, UMR AMAPMontpellier, France; ^2^Instituto de Ecología, Universidad Mayor San AndrésLa Paz, Bolivia; ^3^Departamento de Botánica, Facultad de Ciencias Naturales y Oceanográficas, Universidad de ConcepciónConcepción, Chile; ^4^Instituto de Ecología y BiodiversidadSantiago, Chile; ^5^Institut de Recherche pour le Développement, UR 072, Laboratoire Evolution, Génomes et Spéciation, UPR 9034, Centre National de la Recherche ScientifiqueGif-sur-Yvette, France; ^6^Université Paris-Sud 11Orsay, France

**Keywords:** competition, cushion plants, early snowmelt, facilitation, latitudinal gradient, nurse plant, stress-gradient hypothesis, global warming

## Abstract

While there is a large consensus that plant–plant interactions are a crucial component of the response of plant communities to the effects of climate change, available data remain scarce, particularly in alpine systems. This represents an important obstacle to making consistent predictions about the future of plant communities. Here, we review current knowledge on the effects of climate change on facilitation among alpine plant communities and propose directions for future research. In established alpine communities, while warming seemingly generates a net facilitation release, earlier snowmelt may increase facilitation. Some nurse plants are able to buffer microenvironmental changes in the long term and may ensure the persistence of other alpine plants through local migration events. For communities migrating to higher elevations, facilitation should play an important role in their reorganization because of the harsher environmental conditions. In particular, the absence of efficient nurse plants might slow down upward migration, possibly generating chains of extinction. Facilitation–climate change relationships are expected to shift along latitudinal gradients because (1) the magnitude of warming is predicted to vary along these gradients, and (2) alpine environments are significantly different at low vs. high latitudes. Data on these expected patterns are preliminary and thus need to be tested with further studies on facilitation among plants in alpine environments that have thus far not been considered. From a methodological standpoint, future studies will benefit from the spatial representation of the microclimatic environment of plants to predict their response to climate change. Moreover, the acquisition of long-term data on the dynamics of plant–plant interactions, either through permanent plots or chronosequences of glacial recession, may represent powerful approaches to clarify the relationship between plant interactions and climate change.

## Introduction

Empirical studies and reviews in the last decade leave no doubt that the multiple effects of current and predicted climate change will affect plant communities not only directly, e.g., via “thermophilization,” but also indirectly, through changes in interactions among species (Lortie et al., 2004; Brooker, 2006; Poloczanska et al., 2008; Gilman et al., 2010; Adler et al., 2012; Gottfried et al., 2012; Grassein et al., 2014). To date, however, non-trophic interactions are still poorly considered in predictive models of plant community responses to climate change (Lavergne et al., 2010; Walther, 2010; Bellard et al., 2012; but see Sutherst et al., 2007).

Most efforts aiming at including interactions in predictions of the impact of climate change on plant communities have been based on negative interactions (hereafter termed “competition”; Brooker, 2006; Araújo and Luoto, 2007; Tylianakis et al., 2008; Meier et al., 2012). In comparison, the role of non-trophic positive interactions among species (hereafter termed “facilitation”) in driving the structure and dynamics of plant communities under rapid climate change has rarely been tested, even though conceptual models and reviews predict that this type of interaction might be pivotal, especially in harsh environments (Brooker, 2006; Brooker et al., 2007; Lavergne et al., 2010). Improving our knowledge on facilitation among plants under a changing climate is therefore urgently required as part of assessing the impacts of climate change on plant communities and ecosystems.

The stress-gradient hypothesis (SGH) is one of the most important conceptual advances made over the past two decades with respect to plant–plant interactions along environmental gradients. In its current definition, the SGH predicts that positive interactions among plants (and also among animals: Kawai and Tokeshi, 2007; Dangles et al., 2013) will increase with stress and disturbance, at least up to a certain threshold (Bertness and Callaway, 1994; Brooker and Callaghan, 1998; Michalet et al., 2006; Maestre et al., 2009; He et al., 2013; He and Bertness, 2014). Therefore, a central goal in predicting the response of plant communities in a world affected by climate change is to determine to what extent future environments will modify the levels of disturbance and/or stress experienced by plants (*sensu* Grime, 1977). The direct effects of climate change on plants include warmer temperatures, changes in water availability, and a higher occurrence of extreme events such as drought or heat waves (IPCC, 2013). While limitations related to temperature and water may represent stressors for plants, extreme events are related more to disturbance. Importantly, the effect of a given stress or disturbance level on plants is likely to be site- and species-specific. For example, in dry, warm environments such as the Sahara, warming will certainly increase the environmental stress on plants by increasing evapotranspiration (e.g., Johnson et al., 2012), hence decreasing the water availability (McCluney et al., 2012). Conversely, warming in alpine environments is expected to reduce the stress experienced by plants, thus potentially increasing plant productivity (e.g., Cavieres and Sierra-Almeida, 2012). However, the sum of different stresses or disturbances along environmental gradients will not necessarily generate more facilitation, as demonstrated by the possible existence of non-additive models of interactions (Malkinson and Tielbörger, 2010).

Alpine regions represent an important model for examining the effects of climate change on plant–plant interactions for several reasons. First, alpine ecosystems are relatively homogenous in terms of climatic conditions, and they are found on all continents at almost all latitudes, from 0 to 6000 m a.s.l. (Körner, 2003; Nagy and Grabherr, 2009). Alpine plant communities have been widely used by ecologists over the last two decades to infer patterns and mechanisms of plant–plant interactions, in particular because mountain environments provide abrupt stress variations along elevation gradients (Körner, 2007). Studies conducted in these environments have provided major contributions to the definition and further refinements of the SGH (Choler et al., 2001; Callaway et al., 2002; Maestre et al., 2009; He et al., 2013). In most alpine environments, greater facilitation is observed at higher elevation, i.e., in more stressful conditions—readers are referred to the specific cases in dry alpine environments reported by Cavieres et al. (2006) and Michalet et al. (2014) where two opposing stress gradients were found. Accordingly, these environments constitute a sound model for inferring the effects of climate change on the outcomes of plant–plant interactions.

In this paper, we provide an overview of the extent to which facilitation among plants will interact with the effects of climate change in the organization of alpine plant communities in future decades. We provide a review of current knowledge, and suggest directions for future research. In particular, we focus our contribution on the following four issues and associated hypotheses:

1. *Facilitation in established alpine communities*. Our underlying hypothesis is that decreasing abiotic stress through warming reduces the frequency of facilitative effects among plants that are already established in alpine ecosystems.
2. *Facilitation in alpine communities migrating to higher elevations*. We hypothesize that alpine plants migrating to higher elevations as a response to the effects of warming will interact more positively because of the harsher environmental conditions in the newly settled areas (primary succession).
3. *Facilitation along latitudinal gradients*. We hypothesize that the outcomes of plant–plant interactions will change along large latitudinal gradients, together with the magnitude of climate change and characteristics of the alpine environment.
4. *Facilitation and the long-term buffering effect of alpine nurse plants*. Our hypothesis here is that the persistent buffering effects on microenvironmental conditions by some alpine nurse plants may offer long-term biotic refuges for other alpine plants.

## Facilitation in established alpine communities: a bibliographic review

On 3 April 2014 we conducted a search of the peer-reviewed literature via Web of Science using the following terms: “plants” AND (“alpine” OR “arctic”) AND “climate change” AND (“facilitation” OR “positive interaction”). We obtained a total of 80 references. We then extended this selection by reviewing the relevant literature cited in each of these 80 papers and obtained a second list of 96 references. Later, we reduced this list by retaining only those references that (1) provided explicit data on both climate change and facilitation among plants, and (2) considered facilitation above the treeline (thus excluding forests, but taking into account small and dwarf shrubs). Studies along elevational gradients were only considered if they focussed explicitly on climate change effects. Studies that explicitly documented plant–lichen interactions in the face of climate change were also included. This search resulted in a shortlist of only 17 papers, published between 1997 and 2014 (Table 1). To analyse the data, we considered five parameters: the geographical location of studies; the type of climate change effect (warming, snowmelt timing, water availability); the methodology (experimental, observational, modeling); the number of interacting plants (we assumed that studies involving up to four beneficiary species were “species-pair” studies, in contrast to studies at the community level); and the net interaction outcome (more or less facilitation, neutral, or complex effects with no clear trend). We also took into account the effects of three covariables: nutrients, land abandonment, and wind speed. This quantitative method was not used for Sections Facilitation and the Upward Migration of Alpine Species, Facilitation and Climate Change Along Latitudinal Gradients, and Long-term Facilitative Effects by Nurse plants because of the scarcity of available literature.

**Table 1:** **Review of studies examining facilitation among plants in established alpine communities in the face of climate change**.

**References**	**Country/State**	**Environment**	**Effect**	**Covariable**	**Protocol**	**Climate change proxy**	**Interaction**	**Neighbor**	**Target species**	**Net interaction**
Almeida et al., 2013	Ecuador	Tropical alpine	Warming		Observational	Elevation gradient	Species pairs	*Azorella aretioides*	*Lasiocephalus ovatus*	Facilitation decrease
Brooker et al., 2007	Global	Global	Warming		Modeling	Spatial model	Community scale	Mutualists and competitors	Mutualists and competitors	Complex
Cavieres and Sierra-Almeida, 2012	Chile	Dry alpine	Warming		Experimental	OTC	Species pairs	*Azorella madreporica*	*Hordeum comosum*	Facilitation decrease
Cornelissen et al., 2001	Global	Arctic	Warming	Nutrient	Experimental/Observational	Various	Community scale	Vascular plants	Lichens	Facilitation decrease
Crabtree and Ellis, 2010	Scotland	Alpine	Warming	Wind	Observational	Elevation gradient	Community scale	Vegetation structure	11 lichen species	Complex
Dormann et al., 2004	Norway	Arctic	Warming	Nutrient	Experimental	OTC	Species pairs	*Luzula confusa, Salix polaris*	*Luzula confusa, Salix polaris*	Facilitation decrease
Hobbie et al., 1999	USA/Alaska	Arctic	Warming		Experimental	OTC	Community scale	Seven species	Community	Neutral
Hülber et al., 2011	Austria	Subalpine	Snowmelt timing		Observational	Snowbed-grassland ecotone	Species pairs	Snowbed community	Four snowbed species	Complex
Klanderud and Totland, 2005	Norway	Alpine	Warming	Nutrient	Experimental	OTC	Species pairs	Vegetation structure	*Thalictrum alpinum, Carex vaginata*	Complex
Klanderud and Totland, 2007	Norway	Alpine	Warming		Experimental	OTC	Community scale	*Dryas octopetala*	Seeds of 27 alpine species	Facilitation decrease
Klanderud, 2005	Norway	Alpine	Warming	Nutrient	Experimental	OTC	Species pairs	*Dryas octopetala*	*Thalictrum alpinum, Carex vaginata*	Facilitation decrease
Michalet et al., 2014	Global	Alpine	Warming, water		Observational	Elevation gradient	Community scale	Various	Various	Complex
Olsen and Klanderud, 2014	Norway	Alpine	Warming		Experimental	OTC	Community scale	*Dryas octopetala*	Seeds of 27 alpine species	Facilitation decrease
Pajunen et al., 2011	Finland, Russia	Arctic	Warming		Observational	Shrub abundance	Community scale	Shrubs	Plant community (functional groups)	Complex
Shevtsova et al., 1997	Finland	Subarctic	Warming, water		Experimental	OTC, water addition	Species pairs	*Empetrum nigrum, Vaccinium vitis-idae*	*Empetrum nigrum, Vaccinium vitis-idae*	Complex
Vittoz et al., 2009	Switzerland	Subalpine	Warming	Land abandonment	Observational	Permanent plots	Community scale	Community	Community	Facilitation decrease
Wipf et al., 2006	USA/Alaska	Subarctic	Snowmelt timing		Experimental	Snow manipulation	Species pairs	*Empetrum nigrum, Vaccinium vitis-idae*	*Empetrum nigrum, Vaccinium vitis-idae*	Facilitation increase

### Various climate changes effects, various interaction outcomes

The majority of studies (88%; Table 1) used temperature warming as a proxy for climate change. Indeed, it is one of the most—if not the most—predictable effects of climate change on alpine environments, either in terms of maximum, minimum, or average values (IPCC, 2013). In the majority of these studies (53% of the warming studies), warming decreased the net effects of facilitation among alpine plants, and they never generated an increase in facilitation. In alpine environments, cold temperatures—especially low extremes—are one of the main physical stresses experienced by plants, despite the fact they are generally well adapted to such conditions (Körner, 2003). The buffering of extreme temperature has been shown to be one of the main mechanisms by which nurse plants facilitate other species in alpine regions (Nyakatya and McGeoch, 2008; Badano and Marquet, 2009), supporting the hypothesis that facilitation in alpine environments is primarily generated by stresses that are not directly related to resource availability (Maestre et al., 2009). Therefore, the reduction of temperature stress alongside global warming is expected to reduce the positive effects of alpine nurse plants, lowering net facilitation among plants in established communities, as predicted by the SGH. Facilitation release is thought to be related to (1) higher abundance/cover of competing species (Cornelissen et al., 2001; Vittoz et al., 2009), and (2) a higher growth rate at the individual scale (taller individuals; Klanderud, 2008; Pajunen et al., 2011).

However, the causal relationship between warming and reduced net facilitation among alpine plants is not clear-cut. In one study, reduced facilitation appeared to partly result from land abandonment, which affected plant cover dynamics (Vittoz et al., 2009); whereas, in another study, net facilitation release was possibly compensated by a reduction in wind speed (Crabtree and Ellis, 2010). Five other studies have revealed complex interaction patterns related to (1) site-specific effects (dry vs. temperate; Michalet et al., 2014), (2) species-specific effects (Shevtsova et al., 1997; Klanderud and Totland, 2005; Brooker et al., 2007; Pajunen et al., 2011), and (3) variation in the performance variable used (Klanderud and Totland, 2005). Taken together, these studies suggest uncertainty remains in terms of to what extent warming will reduce the importance of facilitation in the organization of established alpine plant communities. Facilitation release may be more obvious when taking into account interactions between established alpine species and species migrating from lower vegetation belts, as supported extensively in the literature (e.g., Grabherr et al., 1994; Pauli et al., 2012; Olsen and Klanderud, 2014).

**Figure 1:**
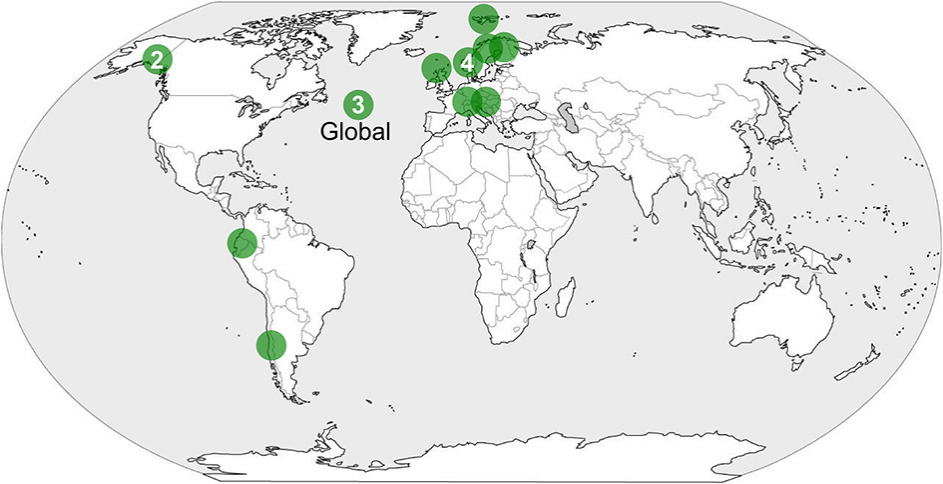
**Location of studies documenting facilitation among plants in established alpine communities under the effects of climate change**.

Greater nutrient availability, in particular through nitrogen deposition, is expected to be another covariable of climate change in alpine regions (Bobbink et al., 1998; Hu et al., 2014) with well-known positive effects on plant productivity (Alatalo and Little, 2014; McDonnell et al., 2014). Interestingly, four studies (Cornelissen et al., 2001; Dormann et al., 2004; Klanderud, 2005; Klanderud and Totland, 2005) found that climate change may interact with changes in nutrient levels, leading to reduced net facilitation among plants (Figure 1). Therefore, it is possible that nutrient enrichment related to nitrogen deposition may be a “hidden” driver of facilitation release in alpine communities also experiencing climate change. This hypothesis is coherent with recent data pointing toward a positive relationship between facilitation and nutrient stress in alpine regions (Yang et al., 2010; Anthelme et al., 2012). These results, supported by a study that took into account nitrogen addition but not warming in the Rocky Mountains (Suding et al., 2008), may question the assumption of Maestre et al. (2009) that plant interactions in alpine regions are not driven by limitations in resources. Nevertheless, the four nutrient enrichment experiments in our review did not specifically simulate nitrogen deposition, but instead used a more complex mixture of nutrients (e.g., *NPK* in Klanderud, 2005; *Hoagland solution* in Dormann et al., 2004). Accordingly, determining the effects of nitrogen fertilization on plant–plant interactions as a consequence of atmospheric contamination remains a challenge.

### Facilitation and early snowmelt, an indirect effect of warming

In temperate-alpine and arctic-alpine regions, snow lasts for several months—except in Equator-facing slopes—such that snowmelt timing has been recognized as a central driver of alpine plant distribution (Körner, 2003; Wipf and Rixen, 2010). An increasing number of empirical studies and reviews have shown that (1) warming diminishes the duration of snow cover, and (2) this reduction is responsible for major changes in the cover, diversity, and productivity of alpine plant communities (Schöb et al., 2009; Wipf and Rixen, 2010; Myers-Smith et al., 2011). Only two studies from our review focussed on the effect of snowmelt timing on plant–plant interactions. By examining survival, phenology, growth and reproduction of two alpine shrubs, Wipf et al. (2006) observed more facilitation between two alpine shrubs with earlier snowmelt. Unlike temperature increase and nutrient enrichment, early snowmelt can be considered as an additional stress as it exposes plants to late freezing events (e.g., Wheeler et al., 2014). Thus, in line with the SGH, facilitative interactions are expected to be more frequent with earlier snowmelt, which was sustained by the data of Wipf et al. (2006). However, this experimental study was carried out over only 1 year. It should therefore be questioned whether or not, under the pervasive effects of warming in the longer term, the frequency of late freezing events (spring frosts) at certain sites will decrease, thereby further conditioning the outcome of interactions (Lavergne et al., 2010). The second study in our review adds complexity to this relationship because it showed that plant–plant interactions were not only influenced by snowmelt timing, but also depended on the performance metrics considered (germination vs. survival of seedlings; Hülber et al., 2011).

### Future research on established alpine communities

Existing data only partially corroborates our first hypothesis that climate change will decrease the frequency of facilitation among alpine/arctic plants. Indeed, the negative effects of warming on observed facilitation may also be the result of co-occurring parameters such as herbivory or wind speed. Moreover, variation in snowmelt timing might compensate facilitation release. Knowledge on the future of plant–plant interactions in established alpine plant communities under climate change is generally scarce. Without such data, even well-conceived conceptual models taking into account plant–plant interactions (e.g., Brooker et al., 2007) may not be able to predict the future of alpine communities under the effects of climate change (Sutherst et al., 2007). Focussing future research on alpine and arctic regions that have thus far been overlooked, including East and North Africa, Papua New Guinea, New Zealand, the Himalaya, Central Asia, Siberia, the Caucasus, the Rocky Mountains, Patagonia, and Antarctica, will certainly add consistency to the hypotheses raised above. Considering not only warming but also focussing on other predictable effects such as snowmelt timing and other environmental changes (herbivory, atmospheric nutrient deposition) is required. Given the fact that extreme events related to temperature or precipitation are expected to occur at a higher frequency in the future, and change the outcomes of plant–plant interactions (e.g., Saccone et al., 2009), the absence of studies taking this factor into account in alpine regions creates bias and requires further research (Wipf et al., 2013). The most challenging climate change effect to study is precipitation, which is difficult to predict given the interplay between global and local factors (Murphy et al., 2004; Loarie et al., 2009).

Another challenging avenue of research for the future is to quantify the cost of being an alpine nurse plant under the effects of climate change. A recent global study in alpine regions evidenced that increasing cover of beneficiary species limited the reproductive output of associated nurse plants (Schöb et al., 2014b), suggesting that a possible increase in the growth rate of alpine beneficiary species because of warming (e.g., Pajunen et al., 2011) may have a negative impact on nurse plants, eventually being a possible cause of population extinction. However, another study observed a reduced negative effect of beneficiary species on nurse plants in more productive ecosystems (Schöb et al., 2014a), possibly indicating that in warmer, more productive environments, negative feedback effects of beneficiary species on nurse plants might diminish. From these seemingly contradictory viewpoints, understanding the feedback effects of beneficiary species on nurse plants in the face of climate change constitutes an important and challenging topic for the future.

**Figure 2:**
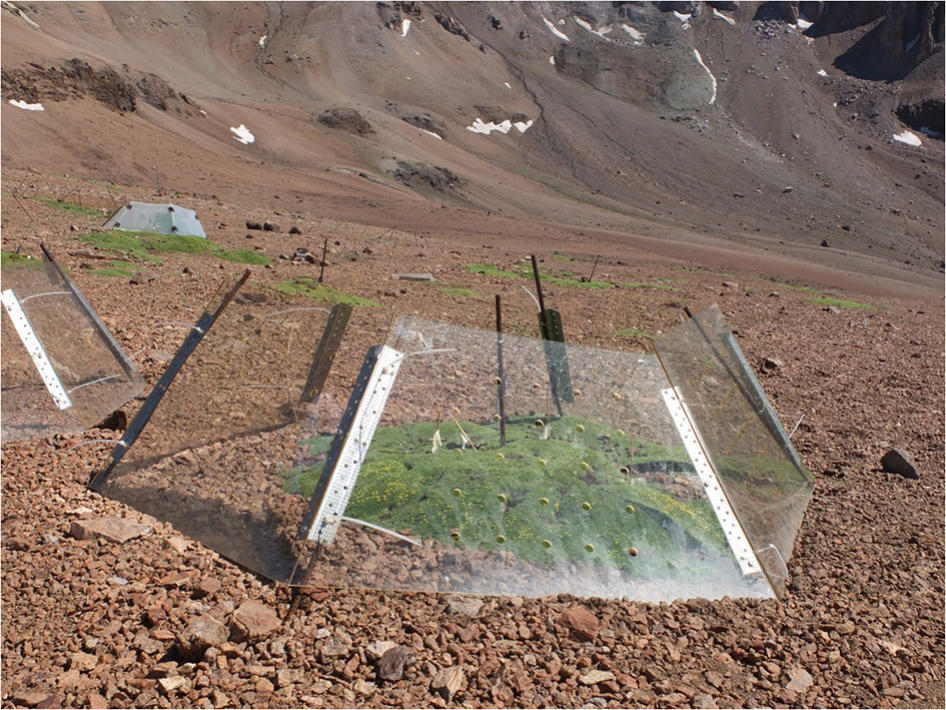
**Experimental site with open top chambers (OTCs) in central Chile**. OTCs were implemented at sites with and without the cushion *Azorella madreporica* with the aim of examining the warming effects on facilitation by *A. madreporica* (Cavieres and Sierra-Almeida, 2012).

From a methodological standpoint, the relatively high proportion of studies on interactions conducted at the community level (53%; Table 1) is encouraging, because the pairwise approach may not provide a representative view of the overall patterns of plant–plant interactions (*species-specific effects*: Cavieres and Badano, 2009; Soliveres and Maestre, 2014). Moreover, the development over the past two decades of open-top chambers (OTC) for experimentally manipulating temperature in alpine-arctic environments has been a crucial development for the study of alpine plant communities (protocol ITEX: Marion et al., 1997; Figure 2). Its use for predicting plant–plant interactions against a background of warming has provided consistent data in alpine and arctic environments (Table 1: 8 studies). It provides a necessary balance between observational and experimental studies, as found in our review (Table 1; Schöb et al., 2012). Other types of manipulative methodological approaches, such as freezing experiments (Martin et al., 2010), CO_2_ enrichment (Hättenschwiler et al., 2002), snow cover manipulation (review in Wipf and Rixen, 2010), ozone concentration and associated UV-B radiation (Searles et al., 2001), and extreme events (Jentsch et al., 2007), should help to develop a more precise conceptual framework of plant–plant interactions by the side effects of climate change in alpine regions.

## Facilitation and the upward migration of alpine species

### Increased stress for plants during accelerated primary succession

**Figure 3:**
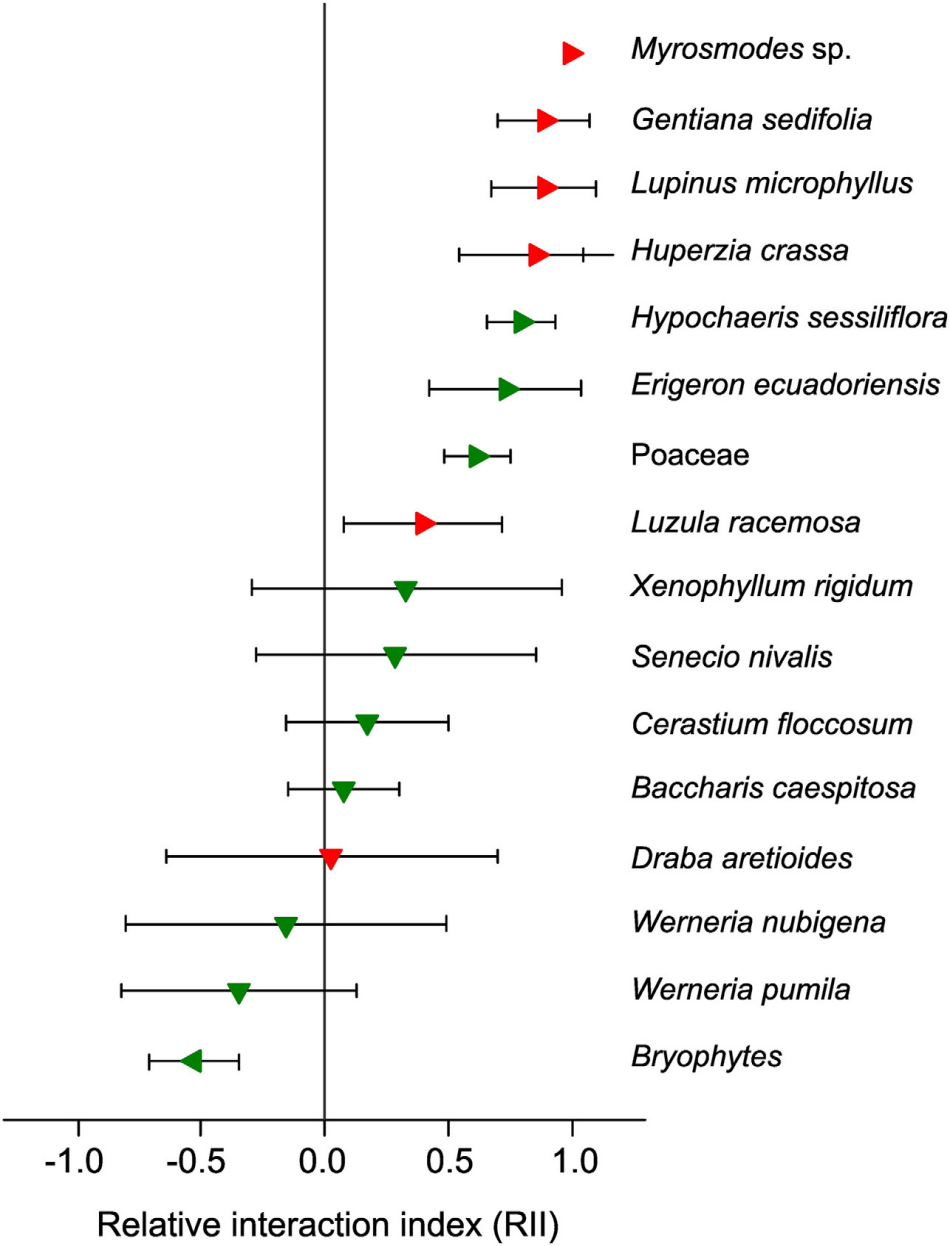
**Tropical alpine species observed in the superpáramo of Volcano Antisana and the outcome of their interactions with the cushion plant *Azorella aretioides* (Ecuador, 4700 m a.s.l.)**. RII > 0: species facilitated by *A. aretioides* (right-facing triangles); RII < 0: species inhibited by *A. aretioides* (left-facing triangles); RII not significantly different from 0: neutral interactions (downward-facing triangles; Anthelme et al., 2012). Red triangles indicate that the species were not observed in the adjacent, recently deglaciated site (between 0 and 13 years after glacial recession; Cauvy-Fraunié, 2010). Green triangles indicate that the species were observed in the recently deglaciated site. Error bars represent the 95% CI.

In the previous section we considered the direct effect of climate change on facilitation in established alpine plant communities. However, it is assumed that the majority of alpine plants are highly sensitive to temperature changes and will exhibit rapid upward migration toward higher elevations (Cannone et al., 2007; Lenoir et al., 2008; Pauli et al., 2012). Such upward migration to previously abiotic areas requires plant adaptations for primary succession, i.e., efficient propagule dispersal and the ability to cope with local environmental filters (Matthews, 1992; Frénot et al., 1998; Walker and del Moral, 2003; Caccianiga et al., 2006). From a climatic viewpoint, it is unlikely that conditions found in these newly colonized areas would be significantly different from those experienced by alpine plants in their original habitat. In contrast, soil properties are likely to differ, from old, organic alpine soils to a complete absence of soil, which is “the defining characteristic of the first stage of primary succession” (Walker and del Moral, 2003). Indeed, soils in newly colonized areas are expected at best to be mostly mineral in terms of their composition and poorly developed, except in scattered “safe sites” (Körner, 2003), thus increasing the level of stress for plants through the absence of resources (nutrients, water), as well as through reduced temperature buffering effects. Therefore, in line with the SGH, stronger facilitation among plants is expected to occur in these newly colonized areas (Stöcklin and Bäumler, 1996; Niederfriniger Schlag and Erschbamer, 2000; Jones and Henry, 2003; Erschbamer et al., 2008). However, given the rapidity of warming over the last four decades in alpine regions (IPCC, 2013), primary succession is very recent and these new areas display very little vegetation cover, comprising mostly of wind-dispersed species (Matthews, 1992) represented by juvenile individuals, which possess less potential to be nurse plants. For this reason, a number of observations made during the earliest stages of alpine primary succession have demonstrated the absence or low occurrence of nurse plants (e.g., Frénot et al., 1998). In the specific case of recently deglaciated areas, dispersal limitation and the slow growth rate of many alpine nurse plants (Ralph, 1978; Morris and Doak, 1998) may also limit the potential for facilitation among plants. To examine this hypothesis, recent data based on the Relative Interaction Index (RII; Armas et al., 2004) in the tropical high-Andes showed that (1) the cushion-forming species *A. aretioides* facilitated 50% of species in the surrounding alpine plant community at 4700 m a.s.l., and (2) the majority of these facilitated species were not present in a recently deglaciated area (0–13 yrs) directly above this site, where *A. aretioides* itself was absent (Cauvy-Fraunié, 2010; Anthelme et al., 2012; Figure 3). In comparison, seven out of eight species not facilitated by *A. aretioides* at 4700 m were present in the recently deglaciated site. These results suggest that, under the effects of accelerated warming, the absence of an important nurse species in a new alpine area targeted for upward migration will have a negative effect on plant diversity by impeding the establishment of associated plants (e.g., *Myrosmodes* sp., *Lupinus microphyllus*; Figure 3). This upholds the hypothesis that the future of (alpine) biodiversity could be partly dependent on facilitative and mutualistic interactions among organisms, required to avoid “chains of extinction” (Choler et al., 2001; Brooker et al., 2008; Bellard et al., 2012).

### Future research on migrating communities

The few data available on facilitation among upward-migrating plants in alpine communities are in line with our second hypothesis of increasing facilitation in these new alpine areas. Nevertheless, knowledge gaps on plant–plant interactions in future alpine communities establishing at higher elevations are even more obvious than those affecting established alpine plant communities, explaining why we were unable to provide a quantitative review for this section. One of the reasons for this fact may be that experimental designs are more complex to set up in these areas, perhaps requiring soil removal instead of being based solely on the installation of OTCs. However, an interesting alternative could be to manipulate conditions through the addition of soil from lower alpine sites into the new alpine sites at higher elevations to examine the performance of species transplanted from the same lower site in a factorial experiment. Implementing this type of experimentation is a stimulating challenge for future research. Knowledge gaps might also be indebted to the prevalence of a conservationist viewpoint of alpine plant communities, leading us to focus our research on established alpine communities threatened by changes rather than future communities establishing themselves at higher elevations. An interesting option to bridge this gap is to collect data in longer term experiments using permanent plots. This is the approach developed by the GLORIA network in alpine environments on the global scale (http://www.gloria.ac.at/) and it would be particularly relevant to take advantage of these designs to focus on the future of plant–plant interactions. Alternatively, using proglacial chronosequences as a space-for-time substitution approach to the study of climate change effects (Blois et al., 2013) may yield important and precise information on the role played by plant–plant interactions during accelerated upward migration of plants, wherever precise data on glacial retreat are available. Indeed, glacial retreat has been a continuous feature worldwide over the last 30–40 years and is documented precisely and regularly at an increasing number of glacial sites on various continents (Rabatel et al., 2013 and references therein). These data have been widely used to document processes of primary succession (e.g., Matthews, 1992; Erschbamer et al., 2001; Caccianiga et al., 2006). However, they have not explicitly considered changes in the direction and intensity of plant–plant interactions over recent decades.

### Alpine plant communities under climate change: the influence of facilitation

**Figure 4:**
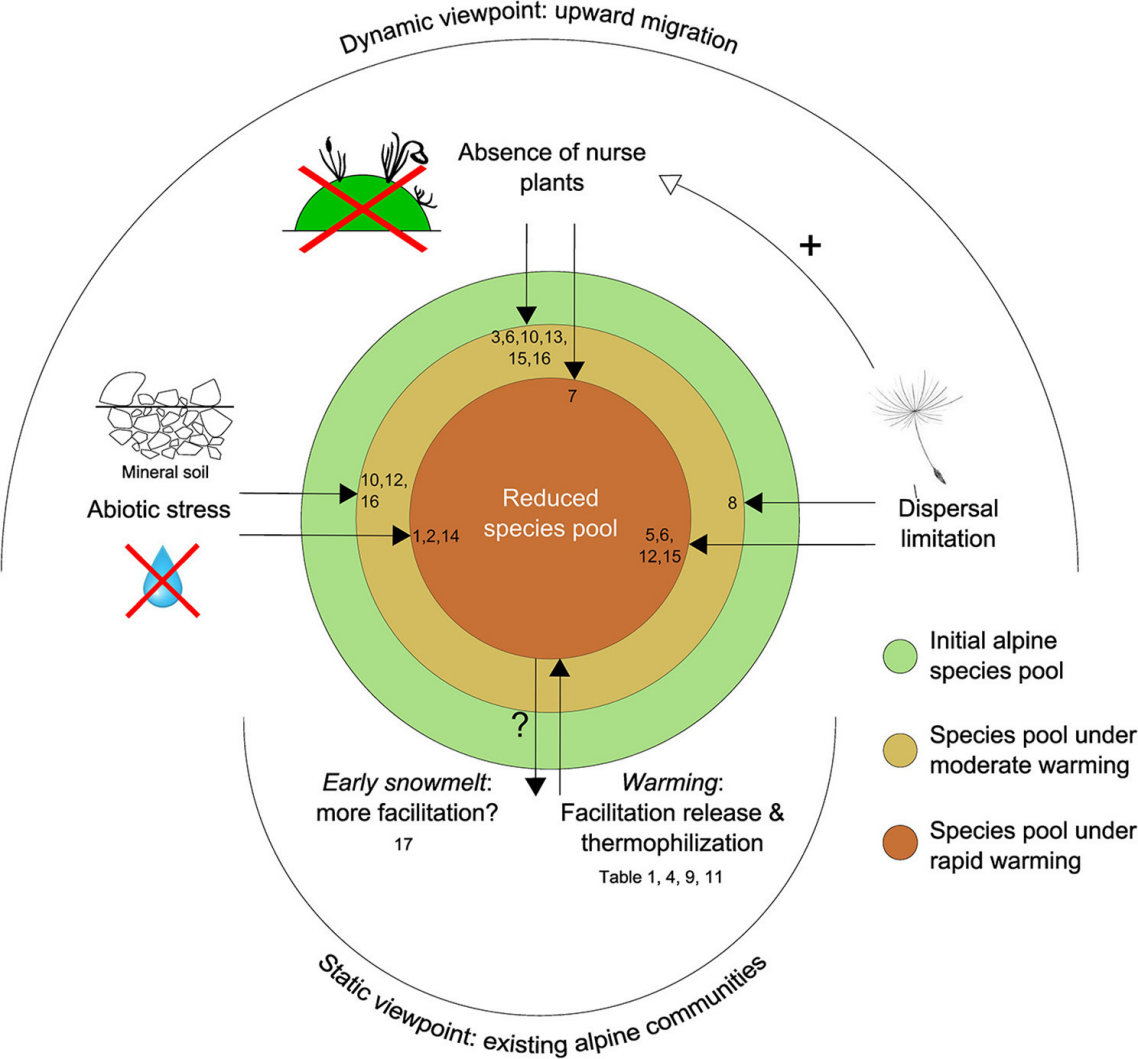
**Facilitation as a driver of alpine community structure under two different paces of warming, in established vs. migrating alpine communities**. References: (1) Anthelme and Dangles, 2012; (2) Caccianiga et al., 2006; (3) Chapin et al., 1994; (4) Chen et al., 2011; (5) Erschbamer et al., 2001; (6) Erschbamer et al., 2008; (7) Frénot et al., 1998; (8) Garbarino et al., 2010; (9) Gilman et al., 2010; (10) Jones and Henry, 2003; (11) La Sorte and Jetz, 2010; (12) Matthews, 1992; (13) Niederfriniger Schlag and Erschbamer, 2000; (14) Sattin et al., 2009; (15) Stöcklin and Bäumler, 1996; (16) Walker and del Moral, 2003 (17) Wipf et al., 2006.

As a global interpretation of Sections Facilitation in Established Alpine Communities: a Bibliographic Review and Facilitation and the Upward Migration of Alpine Species, we propose an exploratory framework to predict the constraints applied to alpine plant communities under global warming, considering both established and migrating plant assemblages, and taking into account the role played by plant–plant interactions (Figure 4). It deals with two different paces of warming. Moderate warming refers to that experienced by Earth between the Little Ice Age (approx. 1650–1750 AD) and the recent acceleration of warming in the 1970s. Rapid, current warming refers to the period from the 1970s to the present day.

In established communities, alpine plants are expected to be impacted negatively by the effects of rapid warming, both directly through thermophilization (Gottfried et al., 2012), but mainly indirectly through net facilitation release (1) with existing neighboring plants (see review in Table 1) and (2) with species migrating from lower elevations (Gilman et al., 2010; La Sorte and Jetz, 2010; Chen et al., 2011). Nevertheless, earlier snowmelt may promote facilitative interactions among plants, at least in the short term (Wipf et al., 2006). At the same time, we do not expect moderate warming to have a significant impact on alpine species pools.

The diversity of upward-migrating alpine communities is expected to be negatively affected by both moderate and rapid warming. Under moderate warming this occurs through dispersal limitation (Garbarino et al., 2010), increasing soil harshness in comparison with the initial alpine site (Matthews, 1992; Stöcklin and Bäumler, 1996; Jones and Henry, 2003), and a deficit in the abundance/maturity of nurse plants (i.e., a deficit in facilitation among plants: Stöcklin and Bäumler, 1996; Niederfriniger Schlag and Erschbamer, 2000; Jones and Henry, 2003; Erschbamer et al., 2008). Moreover, existing data—mostly extracted from the first steps of primary succession after glacial recession—suggest that all these constraints should be exacerbated under rapid warming, i.e., stronger dispersal limitation (Matthews, 1992; Stöcklin and Bäumler, 1996; Erschbamer et al., 2001, 2008), aggravated soil harshness combined with a water availability deficit (Caccianiga et al., 2006; Sattin et al., 2009; Anthelme and Dangles, 2012), and a greater deficiency in the prevalence of nurse plants as a result of a shorter available time for them to recruit and establish in new areas (Frénot et al., 1998).

This overall pattern of alpine species impoverishment is largely dependent on (the absence of) facilitation among plants, thus evidencing the important role that plant–plant interactions may play in the future of alpine communities under the effects of climate change.

## Facilitation and climate change along latitudinal gradients

### Distinctive drivers of interactions at low vs. high latitudes

There are two reasons for taking a closer look at plant–plant interactions along latitudinal gradients when addressing the future of alpine plant communities in the face of climate change: (1) patterns and mechanisms of interactions are expected to vary with latitude; and (2) the intensity of warming may also vary with latitude. A recent review of tropical alpine environments proposed that plant–plant interactions are under the control of a number of drivers, which are distinct from those found in extratropical alpine regions, including aseasonality, the absence of persisting snow cover, different plant life-forms, and the possible inversion of precipitation gradients. They are expected to generate both temperature and water stresses for plants at high elevation sites (Anthelme and Dangles, 2012). As mentioned earlier in the paper, snow cover duration and dry alpine environments are both expected to provide a different outcome with respect to interactions under the influence of climate change (Wipf et al., 2006; Michalet et al., 2014).

**Figure 5:**
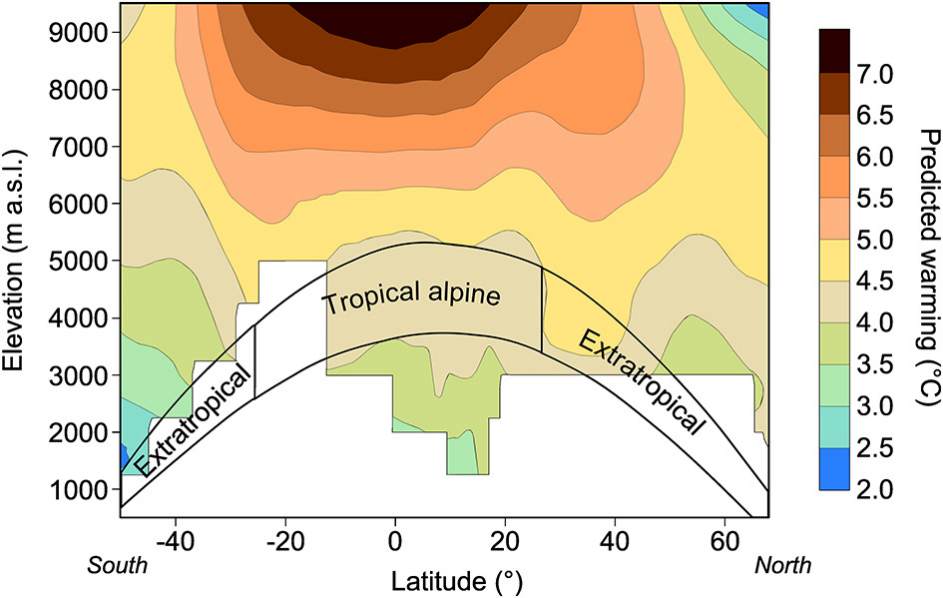
**Projected temperature warming along a latitudinal gradient and an elevation gradient, with a focus on alpine regions, worldwide [from 1990–1999 to 2090–2099, based on the 4th assessment report of the Intergovernmental Panel on Climate Change (IPCC)]**. Modified from Körner (2003) and Bradley et al. (2006).

From a climatic viewpoint, despite the general assumption that future global warming will have a more severe impact in arctic regions (Nogués-Bravo et al., 2007; Loarie et al., 2009; IPCC, 2013), data extracted from wide latitudinal gradients suggest that warming may peak at higher elevations, closer to the troposphere, and at lower latitudes (Bradley et al., 2006; Thompson et al., 2011; Figure 5). This assumption is supported by the greater velocity of glacial retreat observed during the last few decades within the alpine tropics compared to the global scale (Rabatel et al., 2013). When superimposed on the latitudinal distribution of alpine ecosystems, these projections suggest that alpine ecosystems facing the strongest warming in the next few decades will be those distributed at lower latitudes, in the tropics and in the subtropics (Figure 5).

### State-of-the-art and future research on the effects of latitude on facilitation

Existing data on plant–plant interactions along large alpine latitudinal gradients are also scarce, even without considering the effects of climate change. The first global-scale study did not really consider low-latitude sites (mimimum: 26.5°S; Argentina) and did not provide any clear latitudinal pattern (Callaway et al., 2002). Taking advantage of the large latitudinal range of alpine environments in Chile, Cavieres and Badano (2009) used alpine cushions as neighboring species. They evidenced a facilitation peak at 41°S, which decreased—but not regularly—at lower latitudes. More recently, a global study across 78 alpine/arctic sites at all latitudes using cushion plants as neighboring species seemed to confirm this trend, with maximum facilitation observed at moderate- and high-latitude sites, whereas at low-latitude facilitation was found to diminish (Cavieres et al., 2014).

However, the review provided in Section Facilitation in Established Alpine Communities: a Bibliographic Review revealed that there are barely any data on changes in plant interactions with latitude under the effects of climate change. Indeed, most studies have been carried out in temperate and arctic regions (e.g., the Alps, Alaska, Scandinavia), whereas low-latitude alpine regions are largely overlooked. A recent review of nurse plant mechanisms on the global scale lends support to this view by evidencing a strong research gap on plant interactions in tropical (alpine) regions (Filazzola and Lortie, 2014). Only one study has been conducted in tropical alpine environments, supporting the general assumption that more competition will drive established communities, whereas more facilitation will drive the dynamics of upward-migrating communities (Almeida et al., 2013; Figure 4). Accordingly, although an overall pattern of decreasing facilitation with decreasing latitude seems apparent, there are insufficient empirical data to corroborate our third hypothesis that distinct variation in facilitation will be apparent at low and high latitudes under the effects of climate change.

Given the specific environmental charactersitics of low-latitude environments in comparison with high-latitude environments, an important challenge is to provide larger datasets for tropical alpine regions that include climate change designs along the lines of those described in Section Facilitation in Established Alpine Communities: a Bibliographic Review, at several points along large latitudinal gradients [e.g., similar to the gradients studied by glaciologists and climatologists from Alaska to Patagonia (Bradley et al., 2006)]. We expect such an approach to contribute positively to future projections of alpine biodiversity that take into account plant–plant interactions.

## Long-term facilitative effects by nurse plants

### Persistence of buffering effects and alpine life forms

Evaluating the real impact of facilitative processes on plant communities when considering restoration or conservation concerns remains a challenge because of the transient nature of interactions among plants, not only along stress or disturbance gradients, but also along temporal gradients (Bellard et al., 2012; Prévosto et al., 2012). In particular, both nurse plants and beneficiary species can alter the outcome of interactions because of ontogenic shifts (Callaway and Walker, 1997). Several studies have shown that the seedling stage is the best life stage for beneficiary species to be facilitated by nurse plants (see Callaway, 2007 and references therein). However, a recent study in an alpine region indicated that medium-sized individuals rather than seedlings or adults were more prone to facilitation (Le Roux et al., 2013), stressing the need for more explicit studies in this overlooked area (Armas et al., 2013).

**Figure 6:**
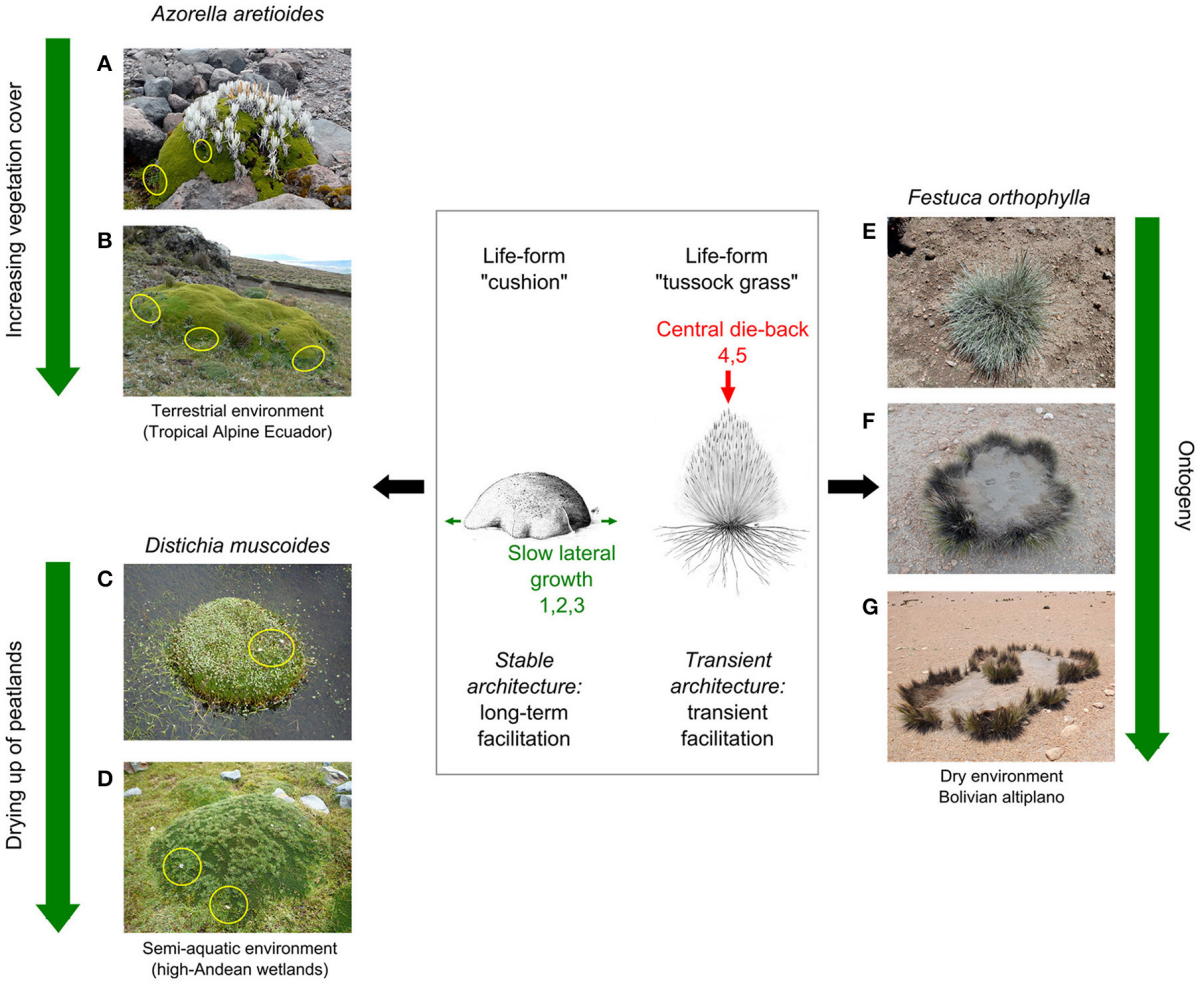
**Two distinct alpine life forms expected to generate two interaction outcomes under the effects of climate change**. On the left, cushion-forming plants: **(A,B)** *Azorella aretioides* in terrestrial environments, with the alpine beneficiary *Hypochaeris sessiliflora* observed both during the first steps of primary succession and long after, when vegetation cover at the soil surface approaches 100% (red circles; Anthelme et al., 2012); **(C,D)** *Distichia muscoides* in semi-aquatic environments, with the beneficiary species *Gentiana sedifolia* (light blue flowers, yellow circles) and *Phylloscirpus deserticola* (light green cover on the cushion) observed even after the area dried up and was colonized by other plant assemblages (Loza Herrera et al., 2014). On the right, the tussock grass *Festuca orthophylla*: **(E)** high stem density in young individuals; **(F)** central die-back in mature individuals; **(G)** intraspecific colonization of the tussock center. (1) Ralph, 1978; (2) Morris and Doak, 1998; (3) le Roux and McGeoch, 2008; (4) Cartenì et al., 2012; (5) Adachi et al., 1996. Drawings: Carlos Maldonado.

In light of the rapidity of climate change affecting alpine regions, the transient nature of interactions is even more puzzling because interacting plants are expected to increase their growth rate (e.g., Hudson et al., 2011), thus modifying substantially the terms of the competition–facilitation equation (e.g., Adler et al., 2012). Nevertheless, many alpine plants develop life forms related to stress-tolerance strategies, i.e., a small size, slow growth rate, and are little sensitive to variations in resource availability (Grime, 1977; Cerabolini et al., 2010). Therefore, we hypothesize that some alpine nurse plants may act as long-term efficient facilitators for other alpine plants under the effects of climate change because their morphology, and thus their buffering effects on the microenvironment, will not change much with time. Two of the most abundant life forms in alpine regions are cushion-forming plants and tussock grasses (Hedberg and Hedberg, 1979; Körner, 2003). Even though tussock grasses have been reported to be highly competitive for resources in dry environments (Gómez-Aparicio, 2009), a number of studies have shown that, within an alpine context, both life forms may act as efficient nurse plants (Callaway et al., 2002; Catorci et al., 2011; Cavieres et al., 2014). We take these two life forms as distinctive cases in the following two paragraphs to explore how the predicted variation in their buffering effects on the microenvironment in the face of climate change may drive their nurse-related impacts on other plants in alpine regions (Figure 6).

Cushion-forming plants (hereafter referred to as “cushions”) comprise 1309 species, the majority of which are alpine species (Aubert et al., 2014). They are slow-growing species that generate biotic substrate, thus “[engineering] their own environment, allowing the system to be less susceptible to direct changes in climate” (Benavides et al., 2013). The relatively regular lateral growth of cushions has enabled reasonably accurate estimations of their age at several hundreds to thousands of years (Ralph, 1978; Morris and Doak, 1998; le Roux and McGeoch, 2004). For these two reasons, cushions provide regular water, buffered temperatures, nutrients and protection from wind for other plants (Cavieres et al., 2002, 2006; Nyakatya and McGeoch, 2008; Badano and Marquet, 2009; Anthelme et al., 2012), i.e., a buffered microenvironment that is expected to be profitable for other alpine plants in the long-term. In the Ecuadorian high Andes, a study on the positive effects of the cushion *Azorella aretioides* on the alpine community showed that the majority of species were facilitated at higher elevation (Figure 6A), whereas neutral or negative interactions prevailed at lower elevation, equivalent to a subalpine, tropical environment (Anthelme et al., 2012). However, even at lower elevation with high vegetation cover at the soil surface, a number of true alpine species such as *Huperzia crassifolia, Hypochaeris sessiliflora, Myrosmodes* sp. or *Oreomyrrhis andicola*—all facilitated at higher elevation—remained present and facilitated by *A. aretioides* (Figure 6B). This suggests that these species take advantage of the longevity and stability of *A. aretioides* to persist at this elevation, which is otherwise colonized by more competitive species from lower elevation, having migrated there because of warming (*Calamagrostis intermedia, Festuca* spp., *Chuquiraga jussieui*). Similarly, in the high-Andean wetlands of Bolivia, the dominant species *Distichia muscoides* provides a terrestrial, but water-saturated substrate for a very specific plant community, which is believed to be threatened by reduced water availability as a result of accelerated glacial recession, a direct consequence of warming (Figure 6C). However, the temporal stability of the cushion's structure and its engineering effect on the environment seems to make it possible for it to persist in the long-term—even if the wetland dries up—thus protecting an entire assemblage of associated plants, such as *Phylloscirpus deserticola* (Figure 6D; Loza Herrera et al., 2014). These two sets of empirical data, although partly speculative, are in line with the hypothesis that the long-term buffering effects of alpine cushions on the microenvironment provide a similarly long-term refuge for true alpine communities that are otherwise unable to cope with increased competition from species migrating from lower elevations.

Along with alpine cushions, tussocks are also long-living life forms (at least several decades; Catorci et al., 2011), but their morphology is much more variable throughout ontogeny. Unlike cushions, tussock stems develop vertically, reducing light and access to the substrate beneath for beneficiary species. Most of all, they frequently experience central die-back in mature individuals, likely because of intra-individual competition for water at their center (Cartenì et al., 2012; Couteron et al., 2014; Figure 6). This ontogenic pattern is cyclic, and new individuals or new ramets colonizing the center of tussocks can themselves experience central die-back, generating complex but structured distribution patterns (Figure 6G). Therefore, unlike young individuals, whose high density of stems make them highly competitive for other plants, mature tussock grasses such as *Festuca orthophylla* (Figures 6E–G) may provide microenvironments with higher nutrient content and reduced negative interactions, thus generating net facilitative effects (Catorci et al., 2011). As a consequence, facilitative effects provided by tussocks are transient, which seemingly does not make this life form a stable biotic refuge for other alpine species trying to escape the effects of climate change.

In summary, these data seem to corroborate our fourth hypothesis that some alpine plants may provide biotic refuges for other alpine species through their long-term, non-transient buffering effects on abiotic parameters in the face of climate change. Cushion plants, whose growth is particularly slow and regular, are examples of such species. Tussock grasses, whose growth is cyclic and irregular, are not.

### Long-term buffering effects and local migration of alpine plants

**Figure 7:**
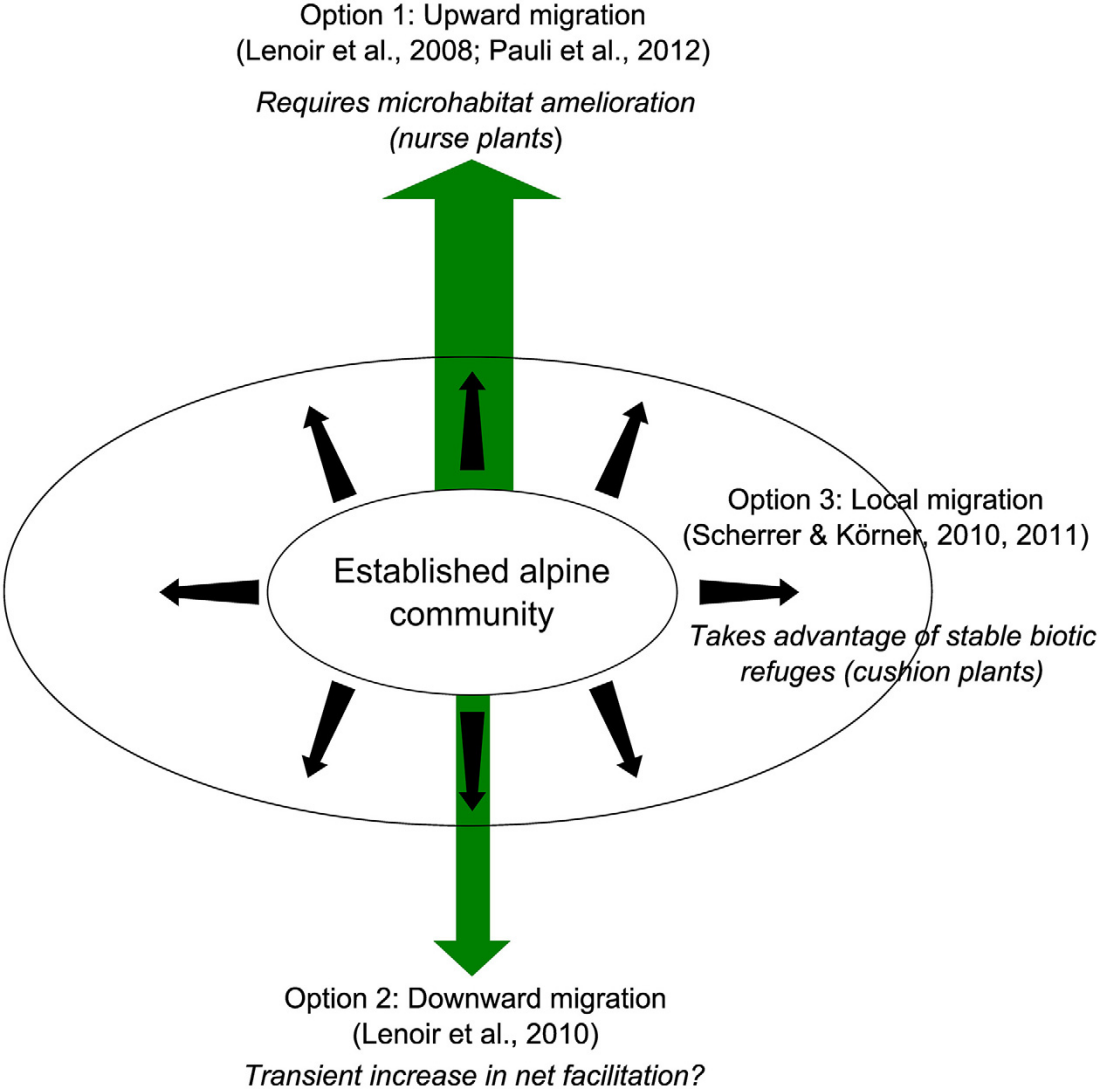
**Three migration scenarios for alpine plant communities under the effects of warming (upward, downward, local) involving three potential facilitative interactions among plants**.

Evidencing the possible long-term facilitative effects of some alpine plants on their neighbors revealed a third migration option for established alpine plants under the effects of climate change, which is directly connected with facilitation among plants. Interestingly, each of these migration options is partially sustained by facilitation among plants (Figure 7). Upward migration is obviously the most widespread pattern observed and requires nurse plants to be successful (e.g., Lenoir et al., 2008; Figures 4, 7). Nevertheless, up to 25% of species may experience significant downward migration, taking advantage of increased disturbance and corresponding increased transient facilitation with other plants in these areas (in line with the SGH; Lenoir et al., 2010). Interestingly, Scherrer and Körner (2010, 2011) identified a third option for alpine plant migration by demonstrating with thermal cameras that local variations in temperature related to microtopography in established alpine communities may exceed IPCC warming projections for the next 100 years. Thus, along with upward and downward migration, local migration may be an important fallback option for alpine plants. The long-term stable architecture of alpine cushions suggests that this local migration may take place not only because of microtopographical variation, but also because of the persistence of such life forms in established alpine communities under global warming, which will permit the presence of other alpine species via facilitative interactions (Figure 7). This assumption agrees with recent data evidencing the pervasive positive effects of alpine cushions on plant communities on a global scale (Cavieres et al., 2014).

### Future research on the long-term facilitative effects of alpine plants

**Figure 8:**
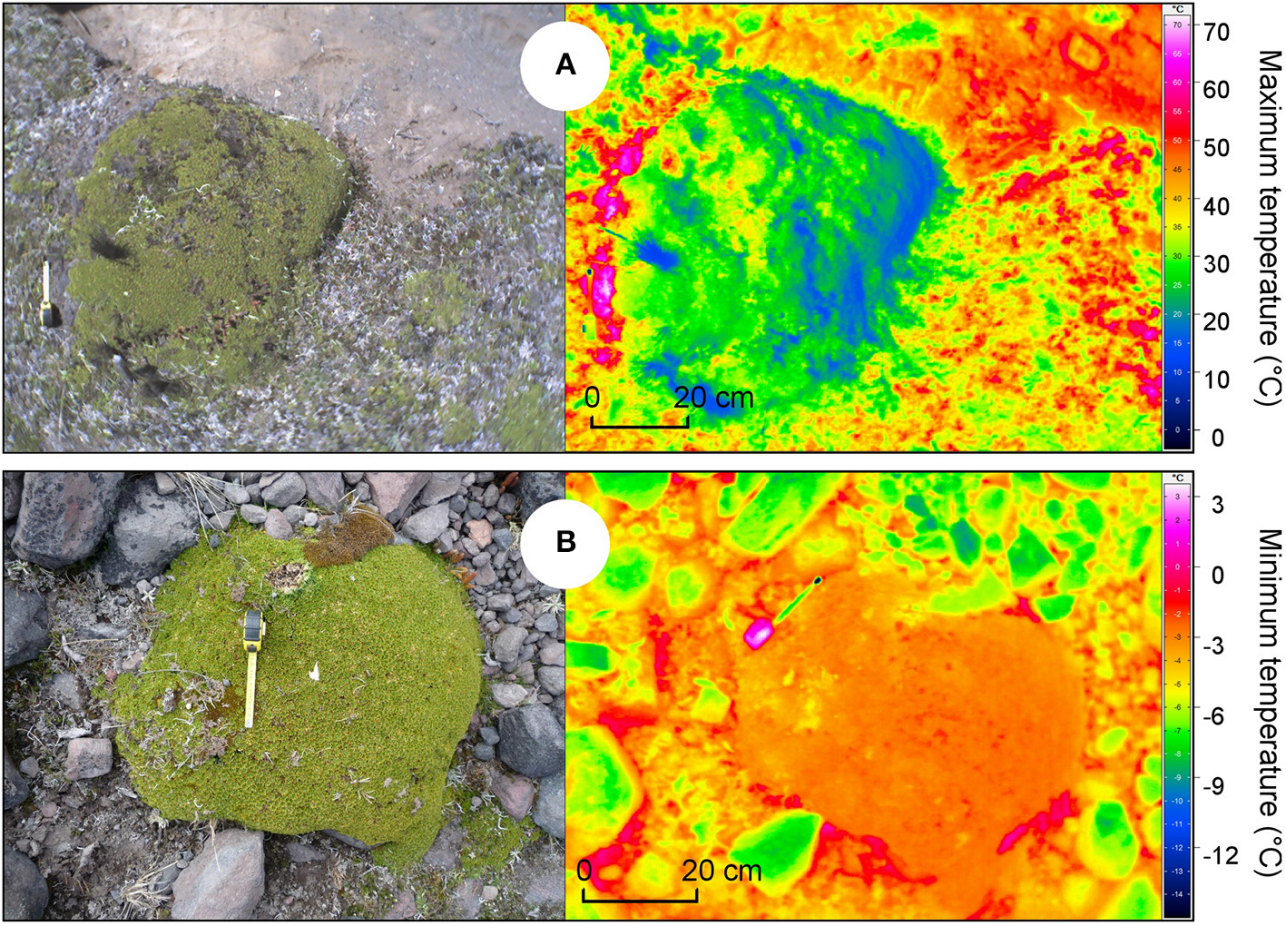
**Spatial representation of the buffering effects of the cushion-forming plant *Azorella aretioides* on temperature (Ecuador, 4700 m a.s.l.)**. **(A)** *A. aretioides* mitigates maximum temperature during the day; **(B)** *A. aretioides* increases minimum temperature at night (unpublished data; Thermographic System VarioCAM® hr—head 680/30 mm GigE).

Temperature buffering has been cited as an important facilitating mechanism by alpine cushion plants. Consequently, the effects of alpine nurse plants on temperature under climate change are predicted to be a crucial driver of the distribution of alpine species. Recent methodological advances in alpine regions have permitted the spatial representation of plant and soil surface temperatures using thermal cameras. Results have demonstrated that variations in surface temperature and root temperature under global warming are not necessarily correlated with atmospheric temperature (Scherrer and Körner, 2010). Accordingly, measuring the surface temperature of potential nurse plants with thermal cameras rather than the air temperature should provide a more accurate explanation for the patterns of observed plant–plant interactions. Repetition of these spatially explicit measurements at different elevations, as a space-for-time substitution of global warming (Blois et al., 2013), may reveal interesting temperature patterns, which—if correlated with structured patterns of plant interactions—could provide an interesting insight into the future direction and intensity of plant–plant interactions in alpine regions. Given the relatively smooth, well-delineated surface of alpine cushion plants, these life forms are expected to be important phytometers for such a purpose. Preliminary results in the High-Andes with the cushion plant *A. aretioides* provide strong agreement with the hypothesis that cushions reduce the maximum temperature during the day, especially in comparison with rocks, bare soil, and dead cushion parts (Figure 8A), and increase the minimum temperature at night, (Figure 8B).

Combining these data with the spatial representation of other abiotic data such as humidity, nutrient availability and topography may provide powerful interpretations of the mechanisms underlying plant–plant interactions at the landscape scale in the face of rapid climate change in alpine regions.

## Author contributions

Fabien Anthelme, Lohengrin A. Cavieres, and Olivier Dangles contributed to the conception, the structure and the writing of the manuscript.

### Conflict of interest statement

The authors declare that the research was conducted in the absence of any commercial or financial relationships that could be construed as a potential conflict of interest.
